# Fungal DNA in pooled equine uterine samples: an exploratory ITS2 amplicon sequencing pilot study

**DOI:** 10.3389/fvets.2026.1978506

**Published:** 2026-10-27

**Authors:** Urtė Pelenė, Rasa Nainienė, Rūta Šveistienė, Inga Merkelytė, Artūras Šiukščius

**Affiliations:** Department of Animal Breeding and Reproduction, Animal Science Institute, Lithuanian University of Health Sciences, Baisogala, Lithuania

**Keywords:** double-guarded swab, fungal culture, fungal DNA, ITS2 amplicon sequencing, low-volume uterine lavage, mare, pregnancy, uterus

## Abstract

Fungal DNA in the equine uterus remains poorly characterized, particularly in mares without overt clinical uterine disease. This exploratory pooled pilot study used ITS2 amplicon sequencing to describe fungal DNA profiles in pooled uterine samples and to perform descriptive comparisons by subsequent pregnancy outcome and sampling method. Conventional microbiological culture findings were summarized as clinical context. samples were collected during naturally occurring estrus and, in mares that were subsequently mated, before the first mating of the studied estrous cycle. Low-volume uterine lavage (LVL) samples from 12 naturally mated Trakehner mares were pooled by outcome into a pregnant-LVL pool (1FR; *n* = 5) and a non-pregnant-LVL pool (2NFR; *n* = 7). The paired sampling-method comparison comprised a paired LVL pool (3LVL) and a paired SWAB pool (4SWAB), each constructed from samples collected from the same five mares. After bioinformatic processing, 82,862–130,870 reads and 27–111 final unique amplicon sequence variants were retained per pooled library. ITS2-target quantitative PCR detected 1,107–1,728 target copies/μL of pooled DNA extract. Neither the extraction blank nor the library-preparation blank crossed the qPCR detection threshold within 40 cycles; however, both yielded sequencing reads, and sequence-resolved control analysis could not exclude low-level laboratory or sequencing-related contamination. A targeted sensitivity analysis excluding the sequence feature shared by the library-preparation blank and two biological libraries did not materially alter the descriptive taxonomic composition, diversity, or ordination patterns. *Ascomycota* predominated across the pooled libraries (50.7–72.5%), followed by *Basidiomycota* (8.1–24.1%). The pregnant-LVL library had the lowest pool-level Shannon diversity value and occupied an ordination position that differed from the other libraries primarily along the first principal coordinate. The paired LVL and swab libraries also differed descriptively in taxonomic composition and ordination position. No fungal growth was reported in the available routine culture reports. These observations demonstrate the presence of diverse fungal DNA signals but do not establish viable uterine colonization, fungal infection, an association with subsequent pregnancy outcome, or equivalence between sampling methods. Confirmation requires sequencing of individual mares, comprehensive field and laboratory controls, standardized mycological culture, and integration with cytological, histopathological, and reproductive data.

## 1 Introduction

Culture-independent studies have expanded understanding of the equine uterine bacterial microbiota and have demonstrated variation among mares, geographical locations, and stages of the estrous cycle (1–4). In other mammalian species, particularly humans, growing evidence indicates that fungi may contribute to microbial interactions and reproductive health within the female reproductive tract, although their biological significance remains incompletely understood (5). More recently, ITS2 amplicon sequencing of pooled uterine lavage samples from postpartum beef cows detected diverse fungal DNA profiles dominated by *Ascomycota*, demonstrating the feasibility of culture-independent uterine fungal profiling in livestock (6). However, differences in host species, reproductive stage, and study design limit extrapolation of those findings to mares. The fungal component of the equine uterine environment remains poorly characterized, and the presence, diversity, viability, and biological relevance of fungal DNA detected in mares without overt clinical uterine disease remain uncertain (7–10).

In equine reproduction, fungi are recognized primarily as opportunistic pathogens associated with endometritis and reproductive failure. *Candida, Aspergillus*, and other yeasts and filamentous fungi have been reported in clinical cases, but most available evidence is derived from culture, cytology, or case reports (8–10). Culture detects viable organisms capable of growth under the applied laboratory conditions, whereas amplicon-based molecular methods detect target DNA and may recover a broader range of fungal assignments. The two approaches therefore provide complementary rather than interchangeable information (7).

Broad-range fungal quantitative PCR followed by sequence analysis has identified fungal DNA in equine endometrial samples in which conventional fungal culture failed to identify a fungal agent (11). More recently, Cheong et al. (7) used next-generation sequencing alongside conventional culture in clinical equine uterine samples and similarly detected fungal DNA assignments that were not recovered by culture. Nevertheless, findings obtained from mares examined for suspected reproductive disease do not establish which fungal DNA signals may occur in mares without overt clinical uterine disease. Moreover, molecular detection alone cannot distinguish viable colonization from transient deposition, non-viable fungal material, or contamination (7, 12, 13).

Two additional questions remain unresolved: whether pooled pre-mating fungal DNA profiles differ descriptively according to subsequent pregnancy outcome and whether LVL and double-guarded uterine swabs recover similar profiles from the same mares. These questions are clinically relevant but require cautious interpretation because equine uterine samples may contain low microbial biomass, making relative-abundance profiles particularly sensitive to sampling procedures, laboratory contamination, DNA extraction, amplification, and bioinformatic processing (12, 13).

The primary objective of this exploratory pooled pilot study was to characterize fungal DNA detected by ITS2 amplicon sequencing in pooled equine uterine samples collected during estrus before the first mating of the sampled cycle. Secondary objectives were to describe pooled-library patterns between mares that subsequently established pregnancy following mating in that cycle and mares that did not establish pregnancy and remained non-pregnant through the end of the 2026 breeding season, to compare paired LVL and double-guarded uterine swab libraries, and to summarize conventional culture findings for the individual samples contributing to the pools. Because each comparison category was represented by a single pooled sequencing library, some mares contributed to both the outcome and sampling-method comparisons, and culture data were incomplete, all comparisons were pre-specified as descriptive and hypothesis-generating; no inferential, causal, or diagnostic claims were intended.

## 2 Materials and methods

### 2.1 Ethical approval

All procedures involving animals were performed in accordance with ethical standards for animal research and complied with the principles of Directive 2010/63/EU of the European Parliament and of the Council on the protection of animals used for scientific purposes. The study protocol was reviewed and approved by the Bioethics Centre of the Lithuanian University of Health Sciences (approval No. 2025-BEC3-T-091).

### 2.2 Animals and study design

This prospective observational study was conducted during the 2026 breeding season at a commercial Trakehner stud farm in Lithuania. A total of 21 Trakehner mares were enrolled. Sixteen mares were bred after sampling and had a recorded pregnancy outcome. Twelve of these mares were naturally mated, and four were artificially inseminated with frozen semen. Overall, seven mares established pregnancy following breeding in the sampled estrous cycle, whereas nine did not and remained non-pregnant through the end of the 2026 breeding season. The remaining five mares were not bred during the sampled estrous cycle.

At the time of sampling, no mare had ultrasonographically detectable intrauterine fluid. Because cytology and histopathology were not performed, the mares are described as having no overt clinical uterine disease rather than as having a clinically healthy endometrium. All samples were collected during naturally occurring estrus. In mares that were subsequently bred, sampling was performed before the first breeding procedure of the studied estrous cycle. Estrous behavior was assessed by teasing with a stallion, and reproductive status was evaluated by transrectal palpation and ultrasonography. Ultrasonographic examination included assessment of ovarian structures, uterine edema, and intrauterine fluid. Uterine edema ranged from grade 1 to grade 3, and no intrauterine fluid was detected at the time of sampling.

Following sample collection, 12 mares were naturally mated and four were artificially inseminated with frozen semen in accordance with the stud farm’s routine reproductive management. Pregnancy was assessed by transrectal ultrasonography approximately 14 days after the final breeding of the studied estrous cycle. Mares were retrospectively classified as pregnant if pregnancy was established following breeding in that cycle, or as non-pregnant if pregnancy was not established and the mare remained non-pregnant through the end of the 2026 breeding season.

Eligibility for the pregnancy-outcome sequencing comparison required a recorded pregnancy outcome and availability of an LVL sample. Twelve mares met these criteria, all of which had been naturally mated. LVL samples from five mares that established pregnancy formed the 1FR pool, whereas LVL samples from seven mares that did not establish pregnancy formed the 2NFR pool. Four additional outcome-classified mares—two pregnant and two non-pregnant—had been artificially inseminated with frozen semen and had swab samples only. These mares were not included in the pregnancy-outcome sequencing comparison because this comparison was restricted to LVL-derived samples, thereby avoiding the combination of different uterine sampling methods within the same outcome-based pools.

Five mares were not bred during the sampled estrous cycle. Four of these mares were enrolled early in the sample-collection period and contributed LVL samples only. Because the paired sampling-method component was initiated after sample collection had already begun, corresponding swab samples were not collected from these four mares. Consequently, they were eligible for neither the pregnancy-outcome comparison nor the paired sampling-method comparison and were not represented in the pooled sequencing libraries. The fifth unbred mare contributed paired LVL and swab samples to the sampling-method comparison. Overall, the paired comparison included five mares, four of which also contributed their LVL samples to 2NFR. Thus, the four pooled sequencing libraries represented 13 unique mares. No enrolled mare was removed from the study cohort. Inclusion in the pooled sequencing comparisons was determined by breeding status and pregnancy outcome, availability of an LVL sample for the pregnancy-outcome comparison, and availability of paired LVL and swab samples for the sampling-method comparison. Consequently, eight enrolled mares were not represented in the pooled sequencing libraries but remained included in the individual-level description of the study population. Individual-level demographic and reproductive characteristics, sampling information, pregnancy outcomes, pooled-library membership, available culture findings, and reasons for non-inclusion in the pooled sequencing libraries for all 21 enrolled mares are provided in Supplementary Table S1.

Because each condition was represented by a single pooled sequencing library, the study was designed to describe pooled fungal DNA profiles and generate hypotheses rather than estimate mare-level associations or causal effects. Characteristics of the mares contributing to the pooled libraries are summarized in Table 1, and the complete pooling structure is presented in Figure 1.

**Table 1:** Characteristics of mares contributing to the pooled ITS2 libraries.

Characteristic	1FR (*n* = 5)	2NFR (*n* = 7)	Paired method comparison 3LVL/4SWAB (*n* = 5)
Age (years), median (range)	7 (4–9)	13 (9–18)	12 (7–17)
Parity, median (range)	2 (0–4)	6 (2–15)	5 (2–7)
Days postpartum at sampling, median (range)	28 (11–55)	791 (28–1,169)	399 (55–1,169)
Uterine edema score, median (range)	2 (1–3)	2 (1–2)	2 (1–2)
Intrauterine fluid present, *n* (%)	0 (0)	0 (0)	0 (0)
Naturally mated, *n* (%)	5 (100)	7 (100)	4 (80)
Not mated, *n* (%)	0 (0)	0 (0)	1 (20)

LVL, low-volume uterine lavage; SWAB, double-guarded uterine swab. Pool labels: 1FR, pregnant-LVL pool; 2NFR, non-pregnant-LVL pool; 3LVL, paired LVL pool; 4SWAB, paired swab pool.

**Figure 1:**
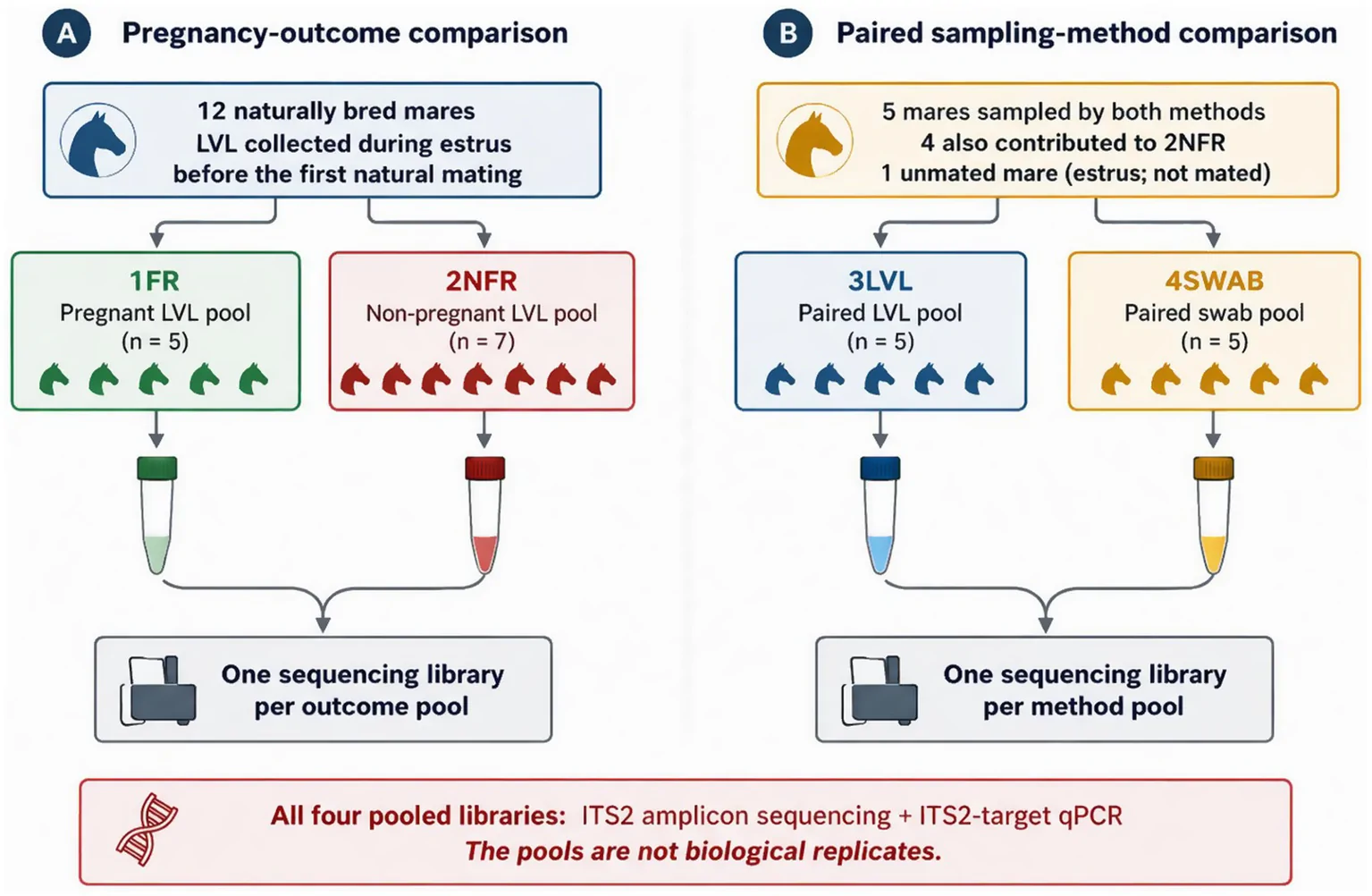
Study design and pooling workflow. Twenty-one mares were enrolled; 16 were bred and had a recorded pregnancy outcome, and five were not bred. **(A)** Pregnancy-outcome comparison. Twelve naturally mated mares with available low-volume uterine lavage (LVL) samples contributed to the pregnancy-outcome pools: five mares that established pregnancy contributed to the pregnant-LVL pool (1FR), and seven mares that did not establish pregnancy contributed to the non-pregnant-LVL pool (2NFR). Four additional outcome-classified mares—two pregnant and two non-pregnant—were artificially inseminated with frozen semen and had swab samples only; they were not included in the pregnancy-outcome sequencing comparison because it was restricted to LVL-derived samples. **(B)** Paired sampling-method comparison. Five mares sampled using both LVL and double-guarded uterine swabs contributed to the paired LVL pool (3LVL) and paired swab pool (4SWAB). Four of these mares also contributed their LVL samples to 2NFR, whereas the fifth mare was not bred. The remaining four unbred mares had LVL samples only and were not included in either comparison because no pregnancy outcome was available and corresponding swab samples had not been collected; the paired sampling-method component was initiated after their samples had been collected. Accordingly, the four pooled sequencing libraries represented 13 unique mares. All four pooled libraries underwent ITS2 amplicon sequencing and ITS2-target quantitative PCR. Each pool generated one sequencing library; n denotes the number of contributing mares rather than replicate libraries.

### 2.3 Uterine sample collection

Samples were collected during estrus. For the 16 mares that were subsequently bred, sampling was performed immediately before the first natural mating or artificial insemination procedure of the studied estrous cycle. The five mares that were not bred during the studied cycle were also sampled during estrus; four contributed LVL samples only, whereas one contributed paired LVL and double-guarded uterine swab samples to the sampling-method comparison. Before sampling, the perineal region was washed with warm water, dried with disposable paper towels, and disinfected with an iodine-based antiseptic solution.

LVL samples were collected from 17 mares, while four additional outcome-classified mares contributed only double-guarded swab samples. A sterile 75-cm equine artificial insemination pipette (Minitube, Tiefenbach, Germany, Ref. 17,209/1175), covered with a hygienic plastic sheath (Minitube, Tiefenbach, Germany, Ref. 17,028/0100), was introduced transcervically into the uterine body. After removal of the protective sheath, 60 mL of sterile 0.9% sodium chloride solution was infused into the uterus and immediately reaspirated through the same pipette without withdrawing the catheter. For molecular analysis, 1 mL of recovered lavage fluid was aseptically transferred with a sterile pipette into a sterile cryovial containing 1 mL of DNA/RNA Shield (Zymo Research, Irvine, CA, USA) and stored at −20 °C until preparation of the biological pools.

For conventional culture of the LVL samples, the entire cotton tip of a sterile swab supplied with Amies transport medium with charcoal (Probact®, TSC Swabs Ltd., Buckingham, UK; TS-5-18) was immersed in the recovered LVL fluid within the collection syringe and rotated to absorb the sample. The swab was then returned to the transport tube, refrigerated immediately after collection, and transported to the diagnostic laboratory within 24 h.

For the sampling-method comparison, paired uterine samples were collected from five mares using both double-guarded uterine swabbing and LVL. To avoid potential alteration of the endometrial surface by the lavage procedure, the double-guarded uterine swabs were collected before LVL. This sampling order was fixed for all five mares and was not randomized. Two separate sterile double-guarded uterine swabs (Minitube, Tiefenbach, Germany, Ref. 17,214/2950) were obtained from each mare. The entire cotton tip of one swab was transferred into a sterile cryovial containing 1 mL of DNA/RNA Shield for ITS2 sequencing, whereas the second swab was placed into Amies transport medium with charcoal for conventional culture. All samples intended for molecular analysis were stored at −20 °C until preparation of the biological pools. Samples intended for culture were refrigerated immediately after collection and transported to the diagnostic laboratory within 24 h.

### 2.4 Microbiological culture and culture-report classification

Routine bacteriological and mycological culture and bacterial isolate identification were performed at the Institute of Microbiology and Virology, Lithuanian University of Health Sciences, Kaunas, Lithuania. Individual LVL-derived transport swabs and directly collected double-guarded uterine swabs were transported under refrigeration in Amies transport medium with charcoal and processed within 24 h of collection. Upon arrival at the laboratory, the transport swabs were streaked directly onto the respective culture media. Because swabs rather than measured liquid aliquots were inoculated, the volume of the inoculum applied to the culture media was not quantified.

For bacteriological examination, specimens were inoculated onto Columbia agar supplemented with 5% sheep blood and incubated aerobically at 37 °C. Bacterial growth was evaluated after 24 and 48 h. Selected specimens were additionally incubated under anaerobic conditions using an AnaeroJar system with a GasPak anaerobic atmosphere generation system (Oxoid, Basingstoke, UK); anaerobic culture was not performed uniformly for all specimens. Morphologically distinct colonies recovered from primary cultures were subcultured onto blood agar until pure bacterial cultures were obtained. Smears prepared from pure cultures were Gram stained when additional characterization of previously unidentified isolates was required.

Morphologically distinct bacterial isolates were identified by matrix-assisted laser desorption/ionization time-of-flight mass spectrometry using a MALDI Biotyper Sirius system (Bruker Daltonics, Bremen, Germany) according to the manufacturer’s routine identification workflow. Biochemical identification systems were not used. Taxonomic identifications included in the study were transcribed from the final diagnostic laboratory reports. Because quantitative colony counts were not consistently available, bacterial growth was summarized descriptively using the reported taxonomic identification and, where applicable, qualitative descriptions of growth.

For mycological examination, specimens were streak-inoculated onto Sabouraud CAF Agar (Liofilchem, Roseto degli Abruzzi, Italy; Ref. 610,203), prepared from a commercially available dehydrated medium. The medium contained enzymatic digest of casein (5.0 g/L), enzymatic digest of animal tissue (5.0 g/L), glucose (40.0 g/L), chloramphenicol (0.5 g/L), and agar (15.0 g/L), with a final pH of 5.6 ± 0.2 at 25 °C. Chloramphenicol was included to suppress bacterial growth. The inoculated plates were incubated in an inverted position under aerobic conditions at 30 °C in darkness and inspected daily for both yeast and mold growth. Cultures were retained for up to 7 days and classified as negative when no fungal growth was observed by the end of the incubation period.

Quality control was performed for the laboratory-prepared medium. For sterility control, one or two uninoculated plates from each prepared batch of 13–26 plates were incubated for 14 days and monitored for microbial growth. Growth-promotion control was performed by inoculating the reference strain *Candida albicans* ATCC 10231 onto the prepared medium and incubating it for 5 days. No growth was observed on the uninoculated sterility-control plates, whereas the *Candida albicans* ATCC 10231 positive control demonstrated the expected growth. No mold reference strain was included as a positive growth control.

All available mycological cultures in the present study were negative; therefore, no fungal isolates were available for microscopic or mass-spectrometric identification. Had mold growth been detected, the laboratory procedure would have included examination of a direct microscopic mount for genus-level morphological identification. Yeast isolates would have been identified by MALDI-TOF mass spectrometry. Antifungal susceptibility testing was not available at the laboratory.

Culture reports were available for 5/5 samples contributing to 1FR, 5/7 samples contributing to 2NFR, 3/5 samples contributing to 3LVL, and 5/5 samples contributing to 4SWAB. Because four LVL samples contributed to both 2NFR and 3LVL, these group-specific counts are not independent. Proportions were calculated using samples with available culture reports as denominators. Missing culture results were not imputed, and no inferential comparisons of culture outcomes were performed.

### 2.5 ITS2 library preparation, sequencing, and bioinformatics

Before submission to the sequencing provider, the biological pools were prepared from the individual DNA/RNA Shield-preserved samples. An equal 1-mL aliquot from each contributing mare was combined without normalization by DNA concentration or ITS2 target concentration. For the LVL-derived pools, 1 mL of the preserved LVL sample from each mare was used. For the swab-derived pool, 1 mL of the DNA/RNA Shield-preserved swab suspension from each mare was used. Sufficient sample volume was available from every contributing mare, and no reduced-volume contributions or other adjustments were required. For the four mares represented in both comparisons, separate 1-mL LVL aliquots were contributed to 2NFR and 3LVL, and a 1-mL aliquot of the corresponding preserved swab suspension was contributed to 4SWAB. This procedure resulted in final pre-extraction pool volumes of 5 mL for 1FR, 7 mL for 2NFR, 5 mL for 3LVL, and 5 mL for 4SWAB. Pool membership was determined according to the pregnancy-outcome and paired sampling-method criteria described in Section 2.2. Four pre-prepared pooled biological samples in DNA/RNA Shield were submitted to the sequencing provider. The pregnant-LVL pool (1FR) consisted of pooled LVL material from five mares that established pregnancy following natural mating in the sampled estrous cycle. The non-pregnant-LVL pool (2NFR) consisted of pooled LVL material from seven mares that did not establish pregnancy following natural mating in the sampled estrous cycle and remained non-pregnant through the end of the 2026 breeding season. The paired LVL pool (3LVL) consisted of pooled LVL material from the five mares included in the sampling-method comparison, whereas the paired swab pool (4SWAB) consisted of pooled swab material from the same five mares. Four mares contributed samples to both 2NFR and the paired 3LVL/4SWAB comparison, whereas one unbred mare contributed only to 3LVL and 4SWAB (Figure 1). Each biological pool generated one sequencing library and therefore did not provide biological replication at the library level. The provider-assigned sample identifiers zr23311-1, zr23311-2, zr23311-3, and zr23311-4 corresponded to 1FR, 2NFR, 3LVL, and 4SWAB, respectively. At the time of revision, the remaining individual samples had already been submitted for separate bacterial 16S rRNA gene sequencing, and no residual individual sample material or extracted DNA suitable for additional ITS2 sequencing remained available. The sequencing provider responsible for the present ITS2 analysis received only the four pre-prepared biological pools and did not retain samples from individual mares. Consequently, retrospective ITS2 sequencing of individual mares could not be performed.

Sequencing was performed by Zymo Research Europe (Freiburg, Germany) using the Microbiome Analysis Service: Targeted Sequencing workflow. The sequencing provider received four pre-prepared pooled samples designated 1FR, 2NFR, 3LVL, and 4SWAB. DNA was separately extracted from 400 μL of each submitted biological pool using the ZymoBIOMICS 96 MagBead DNA Kit (D4308; Zymo Research, Irvine, CA, United States) with 600 μL of Binding Buffer and 8.5 μL of magnetic beads. The manufacturer’s protocol was adapted for automated processing on a KingFisher Flex instrument; according to the sequencing provider, no additional modifications relevant to the reporting of the extraction procedure were made. DNA was eluted in a final volume of 50 μL. Total DNA concentration was measured using the LVIS plate system on a FLUOstar Omega microplate reader (BMG LABTECH). The measured concentrations were 67.70 ng/μL for 1FR, 4.49 ng/μL for 2NFR, 4.33 ng/μL for 3LVL, and 22.28 ng/μL for 4SWAB. ITS2 amplicon libraries were prepared using the Quick-16S NGS Library Prep Kit. The fungal ITS2 region was amplified using the locus-specific forward primer 5′-GCATCGATGAAGAACGCAG-3′ and reverse primer 5′-TCCTCCGCTTATTGATATGC-3′. According to the sequencing provider, the locus-specific primers did not include adapters, linkers, or heterogeneity spacers. Real-time PCR cycle monitoring was used during library preparation to limit chimera formation. Following amplification, the indexed libraries were quantified by qPCR, pooled equimolarly, purified using Select-a-Size DNA Clean & Concentrator, and assessed using TapeStation (Agilent Technologies, Santa Clara, CA, United States) and Qubit fluorometry (Thermo Fisher Scientific, Waltham, MA, USA). The equimolar pooling of amplified libraries was a technical library-preparation step and was distinct from the biological pooling of mare samples performed before submission.

Sequencing was performed on an Illumina NextSeq™ 1,000 platform using a P1 XLEAP-SBS™ Reagent Kit (600 cycles) with a 35% PhiX spike-in. Positive controls consisted of the ZymoBIOMICS Microbial Community Standard and the ZymoBIOMICS Microbial Community DNA Standard for extraction and library preparation, respectively. An extraction blank and a library-preparation blank were included as negative controls. The extraction blank had a post-extraction DNA concentration of 0.338 ng/μL as measured by LVIS. The library-preparation blank was not subjected to DNA extraction; therefore, a post-extraction DNA concentration was not applicable. Both controls underwent library-preparation PCR, were independently indexed, and were included in the final equimolar sequencing pool. Neither control crossed the qPCR detection threshold within 40 amplification cycles; consequently, no post-amplification library concentration or yield could be calculated for either negative control. No field or sampling blank controls were collected. During revision, paired-end FASTQ files and sequence-variant tables were obtained for both negative controls. Sequence variants detected in the negative controls were compared with those in the delivered positive-control and biological-library sequence-variant tables by exact nucleotide-sequence identity. This *post hoc* comparison was performed solely to assess sequence overlap and did not modify the original provider-generated taxonomic composition, diversity, or ordination outputs. No control-informed removal or flagging of sequence features had been applied to the biological libraries in the original provider workflow. After the provider confirmed that the shared feature seq97 had been retained in the original downstream analyses, a targeted sensitivity analysis was performed by excluding seq97 alone, retaining all other sequence features, and rerunning the downstream QIIME analyses. The original provider-generated outputs were retained as the primary descriptive analysis, and the seq97-excluded outputs were used to assess robustness.

Paired-end reads were processed using DADA2 v1.22.0 (14). Primer regions were removed using the DADA2 filterAndTrim function, with 22 bp removed from forward reads and 24 bp from reverse reads. Forward and reverse reads were truncated at 300 and 240 bp, respectively. Quality filtering was performed using maxEE = c(10, 10), truncQ = 2, and rm.phix = TRUE. ASV inference was performed independently for each library without pooled or pseudo-pooled inference. Paired reads were merged using a minimum overlap of 8 bp and a maximum of two mismatches. Chimeras were removed using removeBimeraDenovo with method = “per-sample” and minFoldParentOverAbundance = 5. ASVs represented by ≤10 reads within an individual library were removed; no cross-library prevalence filter was applied. The retained ASVs ranged from 278 to 458 bp, and no upper sequence-length cutoff was applied.

Taxonomy was assigned using UCLUST v1.2.22q, implemented in QIIME v1.9.1 (15, 16), against the sequencing provider’s curated fungal reference database. A minimum sequence identity of 0.90 was required, and up to 200 database hits were evaluated. Hits with identity scores within 0.1 of the highest-scoring hit were retained for consensus assignment. Assignments from phylum to family were accepted only when unanimous across the retained hits; otherwise, the corresponding rank was reported as NA. At the genus level, up to two distinct genera were reported as a hyphenated assignment, whereas assignments involving more than two genera were reported as NA. At the species level, up to three distinct species were reported as a hyphenated assignment; assignments involving more than three species were reported as s__NA. Ambiguous database designations, including unidentified, unknown, uncultured, and sp. entries, were converted to NA. For this independent validation, principal ASVs were operationally defined as those reaching a relative abundance of ≥1% in at least one pooled biological library. These ASVs were queried against the UNITE General FASTA release for Fungi (version 10.0, 19 February 2025) (17) using NCBI BLAST+ v2.17.0 (blastn). Dust filtering and soft masking were disabled, the E-value threshold was set at 1 × 10–10, up to 100 target sequences were retained, and a maximum of one high-scoring segment pair per subject sequence was reported. Hits sharing the maximum bit score were treated as co-best hits, and their conservative lowest common taxonomic assignment was compared with the provider-generated genus and species-level assignments. The composite provider label *Aspergillus–Eurotium* was considered compatible with UNITE *Aspergillus*, and a provider multi-species label was considered concordant when the UNITE assignment matched any of the listed candidate species. This comparison was used solely as an independent taxonomic-concordance assessment and did not replace or recalculate the provider-generated relative-abundance, diversity, heatmap, Bray–Curtis, or PCoA outputs. Detailed ASV-level results are provided in Supplementary Table S3.

The curated Zymo Research fungal reference database is proprietary, internally maintained, hosted on the provider’s internal servers, and not publicly accessible. No formal version identifier, release date, or complete description of the underlying reference sources was available at the time of resubmission. The custom internal script used to calculate the Bray–Curtis distance matrix was not included in the delivered analysis package. The sequencing provider was asked whether further information on the reference database and the custom internal script could be shared externally; however, no additional database documentation or script was available at the time of resubmission. Consequently, exact independent reproduction of the provider-generated taxonomic assignments and Bray–Curtis distance matrix remained limited. The term amplicon sequence variant (ASV) is retained to match the provider’s output. No additional abundance-based filtering was independently applied by the authors. The aggregation of taxa used in Figure 2 was performed solely for visualization and did not alter the underlying sequence-feature data or diversity calculations. Pooling was used as a cost-constrained exploratory strategy to obtain an initial overview of fungal DNA detectable in equine uterine samples. This design eliminated individual mare-level resolution and biological replication; consequently, all sequencing analyses were limited to descriptive, pooled-library-level comparisons.

**Figure 2:**
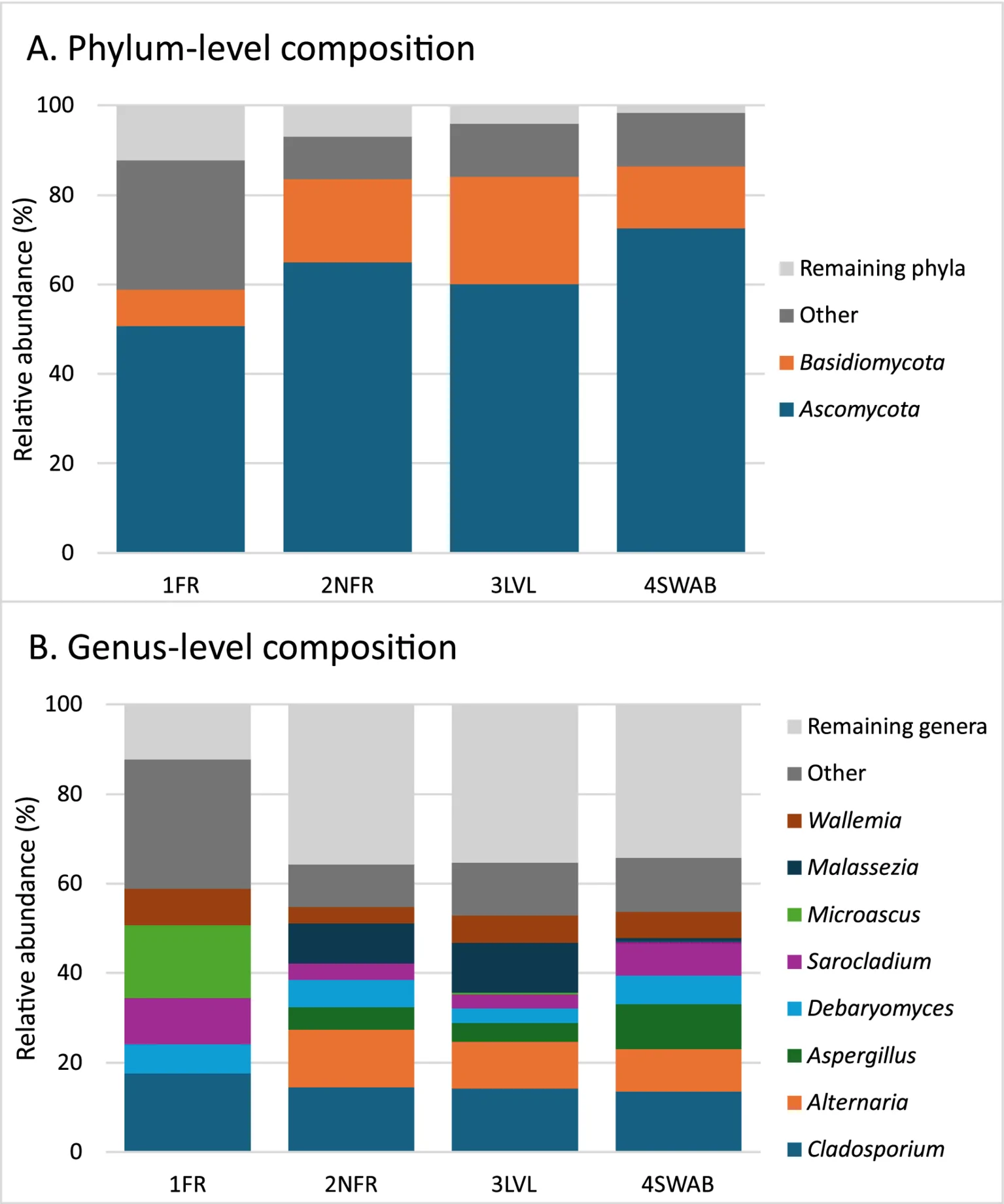
Relative taxonomic composition of fungal DNA in the four pooled ITS2 sequencing libraries. **(A)** Phylum-level relative abundance. *Ascomycota* and *Basidiomycota* are displayed individually, whereas all other named phylum-level assignments are combined as “Remaining phyla.” **(B)** Genus-level relative abundance. The eight genera with the highest mean relative abundance across the four pooled libraries are displayed individually. All other named genus-level assignments are combined as “Remaining genera.” The provider-defined “Other” category represents ASVs for which no match was identified in the curated Zymo Research fungal reference database at the ≥90% sequence-identity threshold. Low-abundance classified taxa were not combined into “Other”; sequences classified only to higher fungal taxonomic ranks retained explicit NA designations.

### 2.6 Quantification of the ITS2 target

ITS2 target concentration was measured by quantitative real-time PCR performed by Zymo Research using 10-fold serial dilutions of plasmid standards containing a single copy of the fungal ITS2 target. Each reaction received 2 μL of DNA extract and used the same ITS2 primers as library preparation. The reported ITS2 standard curve had *R*^2^ = 0.9992. Results were expressed as ITS2 target copies per microliter of DNA extract. No conversion to fungal genome copies or fungal DNA mass was performed because rDNA copy number varies substantially among fungal taxa (18).

### 2.7 Statistical analysis

Because each condition was represented by a single pooled sequencing library, the mare was not retained as the experimental unit for ITS2 analysis, and inferential comparisons were not valid. Alpha-diversity analyses were performed in QIIME v1.9.1. Rarefaction series were generated using parallel_multiple_rarefactions.py with the parameters -m 10 -s 499 -x 20,000. The maximum depth of 20,000 sequences was specified because the smallest biological library contained more than 20,000 final reads. With a starting depth of 10 and an increment of 499, the highest generated depth not exceeding this maximum was 19,970 sequences. Ten random subsampling iterations were performed at each depth without a specified random seed. Alpha-diversity metrics were calculated using parallel_alpha_diversity.py; observed ASV richness and Shannon diversity reported in this study represent the unweighted arithmetic means of iterations 0–9 at a depth of 19,970 sequences. These iterations quantify stochastic subsampling variability and are not biological replicates.

The Bray–Curtis distance matrix was calculated using a provider-maintained custom script from genus-level relative-abundance data in Composition_Summary_L6.txt. The provider-defined unassigned category None; Other; Other; Other; Other; Other was excluded before calculation. The analysis was therefore based on genus-level relative abundances rather than raw or rarefied ASV counts. Principal coordinates analysis was performed using the QIIME v1.9.1 principal_coordinates.py function. For the sensitivity analysis, the provider removed seq97 from the ASV table and reran the taxonomic composition, alpha-diversity, genus-level Bray–Curtis, PCoA, and heatmap workflows using the same reported settings. Conventional culture results were retained at the individual-sample level and summarized as counts and proportions among available reports. No *p* values, confidence intervals, imputation, or claims of association were used; culture proportions were not compared because of small denominators, missing reports, and overlapping LVL membership between 2NFR and 3LVL.

## 3 Results

### 3.1 Sequencing output and quality assessment

ITS2 amplicon sequencing generated data for all four pooled libraries. After trimming, denoising, chimera removal, and final feature filtering, 130,870, 125,023, 119,419, and 82,862 reads were retained for 1FR, 2NFR, 3LVL, and 4SWAB, respectively. The corresponding numbers of final unique ASVs reported by the pipeline were 27, 111, 108, and 71 (Table 2). Neither the extraction blank nor the library-preparation blank crossed the qPCR detection threshold within 40 amplification cycles. The subsequently supplied paired-end FASTQ files contained 1,091 read pairs (2,182 individual reads) for the extraction blank and 2,479 read pairs (4,958 individual reads) for the library-preparation blank. The provider-supplied sequence-variant tables contained one ASV represented by 693 reads in the extraction blank and two ASVs represented by 1,891 and 30 reads, respectively, in the library-preparation blank. The dominant ASV in both negative controls was identical to the dominant positive-control ASV but was absent from the delivered sequence-variant tables for all four biological libraries. The second ASV detected in the library-preparation blank was also present in the delivered sequence-variant tables for 2NFR and 4SWAB, where it was represented by 185 and 533 reads, respectively; it was not detected in the corresponding tables for 1FR or 3LVL. No control-informed removal or flagging of sequence features had been applied to the four biological libraries in the original provider workflow.

**Table 2:** Quality metrics for the four pooled ITS2 libraries.

Metric	1FR	2NFR	3LVL	4SWAB
Raw reads	312,212	299,016	287,396	199,054
Trimmed reads	267,896	257,248	246,378	170,514
Denoised reads	132,951	127,485	121,864	84,418
Non-chimeric reads	130,895	125,080	119,493	82,932
Final retained reads	130,870	125,023	119,419	82,862
Final unique ASVs	27	111	108	71

ASV, amplicon sequence variant; pool labels are defined in Figure 1.

The second ASV (seq97), assigned to *Malassezia restricta*, passed the provider’s chimera, abundance (at least 10 reads per sample), and sequence-length filters and was retained in the original downstream outputs. It accounted for 185 reads (0.148% of the library) in 2NFR and 533 reads (0.643%) in 4SWAB. At the species level, exclusion of seq97 reduced the relative abundance assigned to *M. restricta* from 0.510 to 0.363% in 2NFR and from 0.643 to 0% in 4SWAB. At the genus level, the relative abundance of *Malassezia* decreased from 9.043 to 8.908% in 2NFR and from 0.968 to 0.327% in 4SWAB. Relative abundances of all other taxa changed only marginally because of denominator recalculation following removal of 185 and 533 reads, respectively. After exclusion of seq97, the final read counts were 130,870, 124,838, 119,419, and 82,329, and the final ASV counts were 27, 110, 108, and 70 for 1FR, 2NFR, 3LVL, and 4SWAB, respectively. Mean observed ASV richness values were 26.3, 110.0, 107.5, and 70.0, and mean Shannon diversity values were 3.503, 5.808, 5.718, and 5.502, respectively. PC1 and PC2 explained 66.1 and 25.5% of the variation. The lowest Bray–Curtis dissimilarity remained between 2NFR and 3LVL (0.245 versus 0.244 in the primary analysis), and the greatest remained between 1FR and 3LVL (0.610 in both analyses). Thus, exclusion of seq97 produced only localized changes in the relative abundance of *M. restricta* and *Malassezia* and did not materially alter the overall taxonomic composition, alpha-diversity, Bray–Curtis, or PCoA patterns. Curated provider-generated outputs supporting the sensitivity analysis are provided in Supplementary Data S2.

### 3.2 ITS2 target concentration

The ITS2 target was detected in all four pooled DNA extracts. Concentrations were 1,107 copies/μL in 1FR, 1,258 copies/μL in 2NFR, 1,240 copies/μL in 3LVL, and 1,728 copies/μL in 4SWAB (Table 3). These values quantify the amplified ITS2 target and should not be interpreted as fungal genome counts or viable fungal burden.

**Table 3:** ITS2 target concentration in pooled DNA extracts.

Pool	ITS2 target copies/μL DNA extract
1FR	1,107
2NFR	1,258
3LVL	1,240
4SWAB	1,728

Values represent ITS2 target concentrations measured by plasmid-calibrated qPCR (standard-curve *R*^2^ = 0.9992) and were not converted to fungal genome equivalents.

### 3.3 Conventional culture findings

Routine culture reports were available for 5/5 samples contributing to 1FR, 5/7 samples contributing to 2NFR, 3/5 samples contributing to 3LVL, and 5/5 samples contributing to 4SWAB (Table 4). Named bacterial isolates were reported in 1/5 (20%), 3/5 (60%), 3/3 (100%), and 5/5 (100%) of the available culture reports, respectively. These proportions are descriptive only. No inferential comparisons were performed because of the missing LVL culture reports, the repeated representation of four LVL samples in both 2NFR and 3LVL, and the small number of available samples.

**Table 4:** Conventional culture findings for individual samples represented in the pooled ITS2 libraries.

Pool	Culture reports available, *n*/*N*	Reports with named bacterial isolate(s), *n*/*N* (%)	Fungal growth, *n*/*N* assessed (%)
1FR	5/5	1/5 (20%)	0/5 (0%)
2NFR	5/7	3/5 (60%)	0/5 (0%)
3LVL	3/5	3/3 (100%)	0/3 (0%)
4SWAB	5/5	5/5 (100%)	0/5 (0%)

LVL, low-volume uterine lavage. Denominators represent samples with available culture reports. Four LVL samples contributed to both 2NFR and 3LVL; therefore, these rows are not independent and should not be summed.

The reported bacterial isolates included *Streptococcus equi subsp. zooepidemicus, Streptococcus mitis, Staphylococcus equorum, Staphylococcus capitis, Microbacterium maritypicum, Globicatella sanguinis, Achromobacter insolitus, Corynebacterium uterequi*, and *Paraburkholderia xenovorans*. Several swab culture reports described bacterial growth qualitatively as one colony, very low quantity, or low quantity; quantitative colony counts were not available.

No fungal growth was recorded in any culture report in which the fungal outcome was explicitly documented (Table 4).

Complete paired LVL and swab culture results were available for three mares. In one mare, at least one concordant bacterial taxon was recovered with both sampling methods, whereas the other two mares had different bacterial isolate profiles in their LVL and swab samples. Two additional mares had swab culture results only, precluding complete paired comparisons.

### 3.4 Taxonomic composition

*Ascomycota* was the predominant phylum in every pool, accounting for 50.7%, 64.9%, 60.0%, and 72.5% of assignments in 1FR, 2NFR, 3LVL, and 4SWAB, respectively. *Basidiomycota* represented 8.1%, 18.7%, 24.1%, and 13.9%, respectively (Figure 2A).

*Cladosporium* was detected in all pools at 13.5–17.6% relative abundance. The 1FR profile contained *Microascus* (16.3%), *Sarocladium* (10.3%), *Wallemia* (8.1%), and *Debaryomyces* (6.5%), while *Alternaria, Aspergillus*, and *Malassezia* were not represented among the plotted dominant genera. In 2NFR, *Alternaria* (12.8%) and *Malassezia* (9.0%) were prominent; 3LVL contained *Malassezia* (11.2%) and *Alternaria* (10.4%); and 4SWAB contained *Aspergillus* (10.0%), *Alternaria* (9.5%), and *Sarocladium* (7.5%) (Figure 2B). Fifty-nine ASVs reached a relative abundance of ≥1% in at least one pooled biological library and were included in the independent UNITE comparison. Among 43 principal ASVs with a provider-assigned genus, 34 (79.1%) were concordant with the genus-level lowest common assignment of the co-best UNITE hits, four (9.3%) could not be resolved to a single genus across co-best hits, and five (11.6%) were discordant. Among 34 principal ASVs with a provider-assigned species or candidate-species label, 17 (50.0%) were concordant, six (17.6%) could not be resolved to a single species across co-best hits, and 11 (32.4%) were discordant. Thus, concordance was higher at genus than at species level (Supplementary Table S3).

### 3.5 Alpha diversity

At a rarefaction depth of 19,970 sequences per pooled library, mean observed ASV richness across 10 random subsampling iterations was 26.7 in 1FR, 110.8 in 2NFR, 107.8 in 3LVL, and 71.0 in 4SWAB (Figure 3A). The corresponding mean Shannon diversity values were 3.502, 5.818, 5.710, and 5.525, respectively (Figure 3B). Thus, 1FR had the lowest pool-level richness and Shannon diversity, whereas 2NFR had the highest values for both metrics. The subsampling iterations quantify stochastic variability due to rarefaction and do not represent biological replication or distributions among individual mares. No inferential comparisons were made.

**Figure 3:**
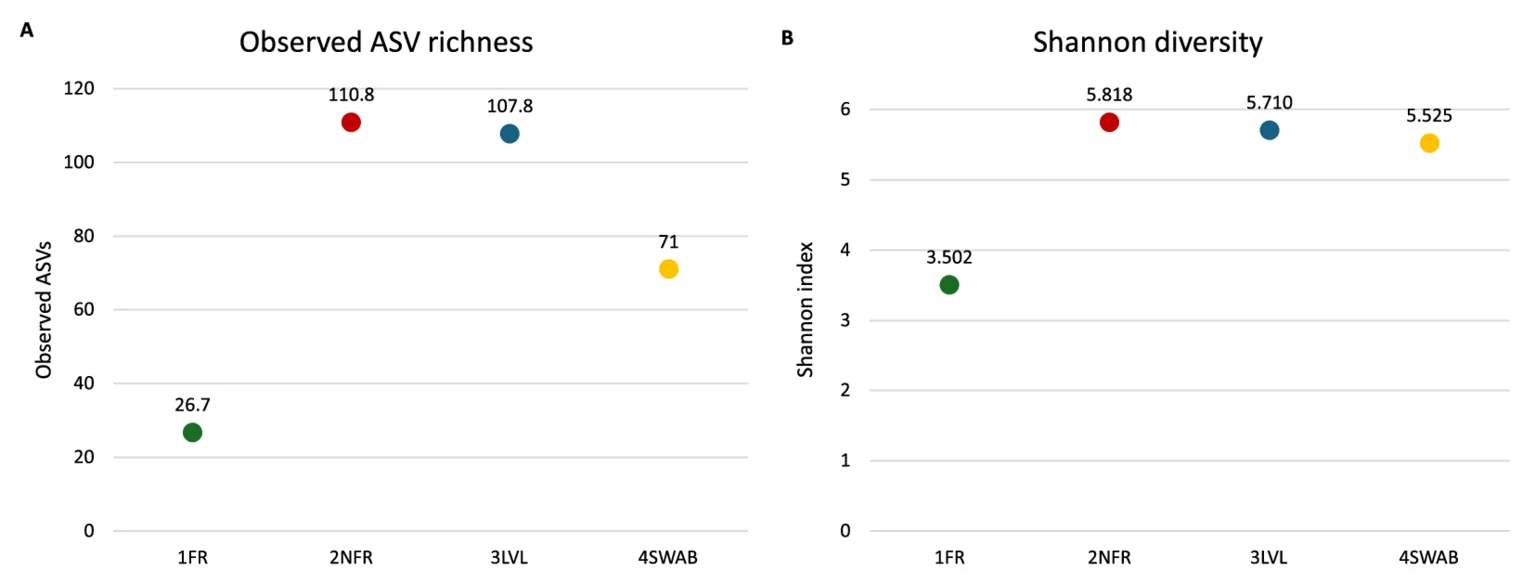
Alpha-diversity metrics for the four pooled ITS2 sequencing libraries at a rarefaction depth of 19,970 sequences per library. **(A)** Mean observed ASV richness. **(B)** Mean Shannon diversity. Values represent the means of 10 random subsampling iterations for each pooled library. The iterations quantify stochastic rarefaction variability and are not biological replicates. No inferential comparisons were made.

### 3.6 Beta diversity

Bray–Curtis PCoA placed the four pooled sequencing libraries at different coordinates in the ordination, with PC1 and PC2 accounting for 66.5 and 25.1% of the total variation, respectively (Figure 4). The pregnant-LVL library (1FR) differed from the other libraries primarily along PC1. The non-pregnant-LVL (2NFR) and paired LVL (3LVL) libraries occupied the most similar positions and had the lowest pairwise Bray–Curtis dissimilarity (0.244). The paired swab library (4SWAB) differed from its paired LVL counterpart (3LVL) primarily along PC2. The greatest pairwise dissimilarity was between 1FR and 3LVL (0.610). These observations describe dissimilarity among four individual pooled libraries. Because each analytical category was represented by a single pooled library, the ordination cannot demonstrate group separation, clustering, or within-group dispersion.

**Figure 4:**
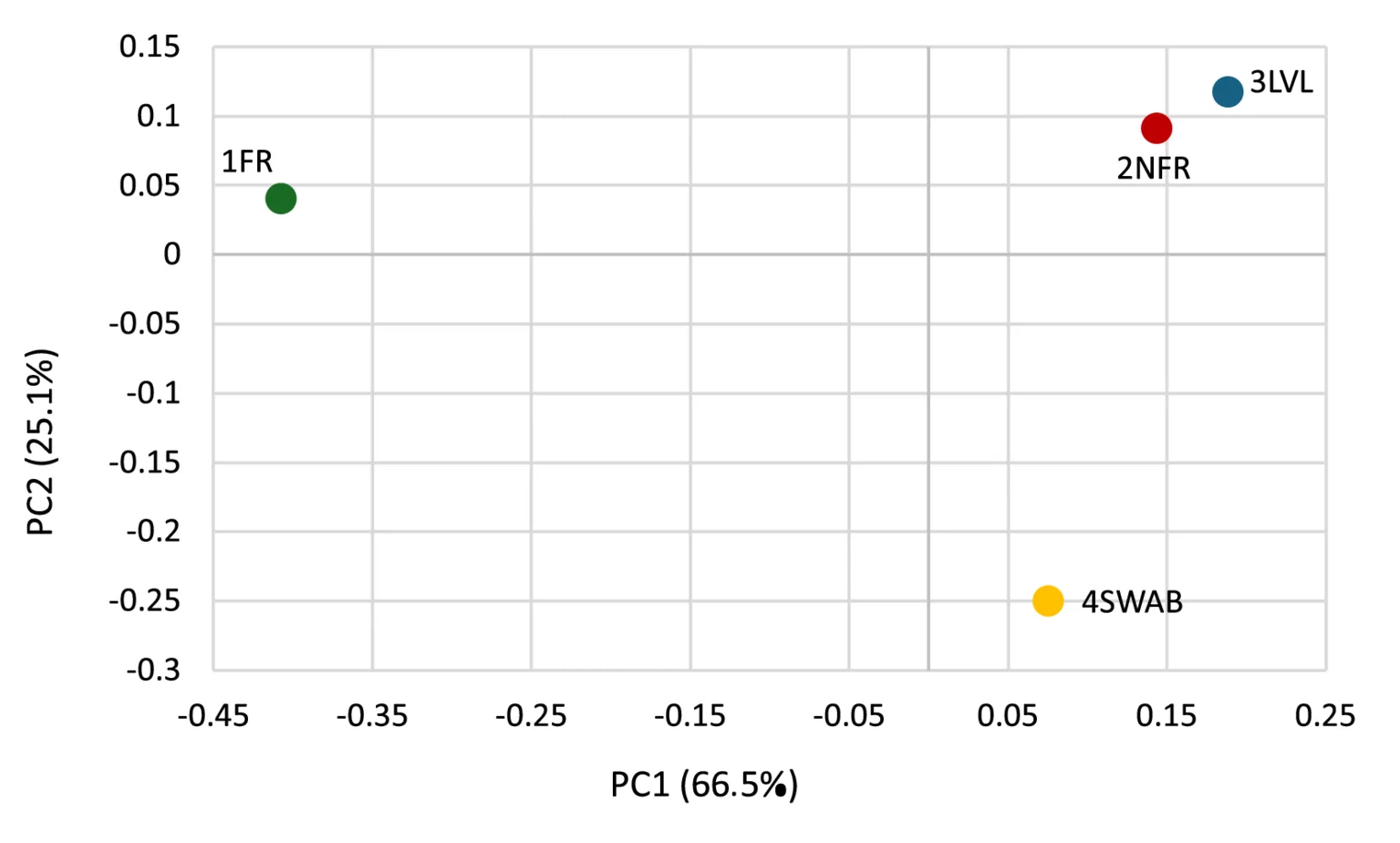
Principal coordinates analysis (PCoA) based on Bray–Curtis dissimilarities among the four pooled-sample fungal ITS2 sequencing libraries. PC1 and PC2 accounted for 66.5 and 25.1% of the total variation, respectively. Each point represents one pooled sequencing library. Because each analytical category was represented by a single library, the observed positions are descriptive and do not demonstrate group separation or clustering; no inferential testing or within-group dispersion analysis was performed.

## 4 Discussion

This exploratory pooled pilot study provides a descriptive account of fungal DNA detected in pooled equine uterine samples collected during naturally occurring estrus. ITS2 targets were detected in all four pooled DNA extracts, and the corresponding sequencing libraries contained a broad range of fungal taxonomic assignments dominated by *Ascomycota* and *Basidiomycota*. The libraries differed descriptively in taxonomic composition, pool-level Shannon diversity, and ordination position. However, these observations represent contrasts among four composite libraries rather than replicated estimates for biological groups or individual mares. The findings therefore demonstrate analytical detection of fungal DNA and identify hypothesis-generating patterns, but they do not establish the presence of a resident or viable uterine mycobiome, a fungal infection, or an association between pre-mating fungal DNA profiles and subsequent pregnancy outcome.

The predominance of *Ascomycota* across all four pooled libraries is consistent with a recent ITS2-based study of pooled uterine lavage samples from postpartum beef cows, in which *Ascomycota* also represented the dominant fungal phylum (6). This similarity shows that ascomycetous fungal DNA can be detected in uterine samples from different livestock species. Nevertheless, it should not be interpreted as evidence of a conserved uterine fungal community. Differences in host species, reproductive status, sampling period, sample processing, sequencing procedures, and pooling strategy preclude direct biological comparison.

The detection of *Cladosporium, Alternaria, Aspergillus, Sarocladium, Microascus, Malassezia, Wallemia*, and *Debaryomyces* is compatible with the broad taxonomic reach of ITS2 sequencing. Some of these genera include opportunistic fungi reported in equine reproductive disease (8–10). However, their detection in a uterine sample does not demonstrate viability, metabolic activity, endometrial adherence, inflammation, or pathogenicity. This distinction is especially important because agreement between fungal culture and sequencing in clinical equine uterine samples is incomplete (7).

Conventional culture provided individual-sample clinical context but did not directly validate the pooled ITS2 profiles. Bacteria were recovered from some LVL and swab samples, including *S. equi subsp. zooepidemicus*, whereas no fungal growth was observed in the available mycological cultures. The mycological cultures were performed using chloramphenicol-containing Sabouraud CAF Agar, incubated aerobically at 30 °C in darkness, and inspected daily for up to 7 days. The medium batch met the applied sterility and yeast growth-promotion quality-control criteria. Nevertheless, the negative findings indicate only that no fungal growth was detected under the applied culture conditions. They do not exclude viable fungi present at low abundance, slow-growing fungi requiring longer incubation, or fungi requiring different nutritional or atmospheric conditions. The absence of fungal growth is therefore not inconsistent with ITS2 detection because culture assesses cultivability under the applied conditions, whereas amplicon sequencing detects target DNA and does not establish viability (7, 11). Ferris et al. (11) identified fungal agents in all 12 diagnostic equine endometrial samples evaluated using broad-range qPCR followed by sequence analysis, whereas conventional fungal culture identified a fungal agent in only 8 samples. Nevertheless, because those samples originated from diagnostic submissions, their findings cannot be used to infer the presence of viable fungi or fungal disease in the mares without overt clinical uterine disease included in the present study. Furthermore, without cytology or histopathology, neither fungal DNA detection nor bacterial isolation establishes endometritis (19).

All five available swab cultures and all three available paired LVL cultures yielded named bacterial isolates, but only three mares had culture results from both sampling methods and isolate overlap occurred in one. This incomplete pattern cannot establish superior bacterial recovery by either method because two LVL cultures were missing, only three complete pairs were available, and several swab isolates were reported only in one colony or low quantity. Culture results should therefore be interpreted as ancillary descriptive findings rather than evidence of diagnostic performance.

Culture findings from LVL and directly collected uterine swabs were not interpreted as a quantitative comparison of sampling methods. For LVL culture, a transport swab was immersed in the recovered lavage fluid, whereas the double-guarded uterine swab represented direct endometrial sampling. Because the amount of LVL absorbed by the transport swab was not quantified and quantitative colony counts were not consistently available, differences in culture recovery could reflect both the uterine sampling procedure and the subsequent inoculation workflow.

The 1FR and 2NFR libraries differed descriptively in ASV richness, Shannon diversity, taxonomic composition, and ordination position. The pregnant-LVL library contained fewer reported ASVs, had a lower pool-level Shannon value, and occupied an ordination position that differed from the other libraries primarily along PC1. Because samples were collected before the first mating of the studied estrous cycle, these observations represent differences between two pooled pre-mating fungal DNA profiles constructed according to subsequently recorded pregnancy outcome. They do not demonstrate that fungal diversity or any detected taxon influenced pregnancy establishment or reliably distinguished mares that subsequently became pregnant from those that remained non-pregnant.

Interpretation is further limited by the absence of biological replication and imbalance in host characteristics. Each outcome was represented by one composite sequencing library, and pooling eliminated individual-mare variation and prevented estimation of within-group variability, taxon prevalence, effect size, uncertainty, and confounder-adjusted associations. Mares contributing to 1FR were younger, had lower parity, and had a shorter postpartum interval than those contributing to 2NFR (Table 1). In mares, advancing age and multiparity are associated with impaired uterine clearance and increased susceptibility to persistent breeding-induced endometritis (20). Although these relationships do not demonstrate an effect on uterine fungal DNA composition, they indicate that differences in age and reproductive history are biologically relevant potential confounders. Host characteristics may influence reproductive-tract fungal profiles; in cattle, parity and reproductive performance have been associated with cervicovaginal fungal community structure (21). However, comparable evidence for the equine uterus is currently lacking. Consequently, the observed library-level differences cannot be attributed specifically to subsequent pregnancy outcome.

The comparison between 3LVL and 4SWAB was also descriptive. These libraries were constructed from paired samples obtained from the same five mares, thereby controlling mare membership at the pooling stage. However, pooling eliminated the ability to analyse the five mare-level pairs and produced only one LVL and one swab sequencing library. The swab-derived library had the highest ITS2 target concentration and differed from the paired LVL library in taxonomic composition and ordination. These differences may reflect the uterine area sampled, contact with the endometrial surface, recovered biomass, dilution inherent to lavage, sample handling, method-specific contamination, or the fixed sampling order. Because the swab was consistently collected before LVL, a potential sampling-order effect cannot be separated from the effect of sampling method. Therefore, the observed differences do not establish superiority, equivalence, diagnostic agreement, or interchangeability between LVL and double-guarded uterine swabbing.

Low-biomass microbiome studies are particularly sensitive to DNA introduced by reagents, consumables, the sampling environment, index misassignment, and cross-contamination (12, 13, 22). Both the extraction blank and the library-preparation blank remained below the qPCR detection threshold throughout 40 amplification cycles; nevertheless, sequencing reads and sequence variants were recovered from both controls. The dominant ASV in both negative controls was assigned to *Saccharomyces cerevisiae* and was identical to the dominant positive-control ASV but was absent from the final sequence-variant tables for the four biological libraries. This pattern was considered compatible with low-level index misassignment or sequencing cross-talk from the positive control, although the underlying mechanism could not be established conclusively. A second ASV, seq97, was detected at 30 reads in the library-preparation blank and was also present in 2NFR and 4SWAB. The provider assigned seq97 to *Malassezia restricta* and confirmed that it had passed chimera removal, the minimum-abundance threshold of 10 reads per sample, and sequence-length filtering and had therefore contributed to the original taxonomic composition, diversity, heatmap, Bray–Curtis, and PCoA outputs. In the original biological libraries, seq97 accounted for 185 reads (0.148%) in 2NFR and 533 reads (0.643%) in 4SWAB. Exclusion of seq97 reduced the relative abundance assigned to *M. restricta* from 0.510 to 0.363% in 2NFR and from 0.643 to 0% in 4SWAB. At the genus level, the relative abundance of *Malassezia* decreased from 9.043 to 8.908% in 2NFR and from 0.968 to 0.327% in 4SWAB. The targeted sensitivity analysis did not materially alter the overall taxonomic composition, Shannon diversity, Bray–Curtis, or PCoA patterns. Because the direction of transfer cannot be determined, the shared feature could represent low-level technical contamination or transfer from a genuine biological signal into the blank. The original outputs were therefore retained as the primary analysis, with the seq97-excluded outputs reported as a sensitivity analysis. In addition, no field or sampling blank controls were collected, preventing assessment of contamination introduced during sample collection and initial handling. Consequently, the uterine origin of all detected fungal DNA signals cannot be established conclusively.

ITS2 amplicon data also have marker-specific limitations. Primer affinity, amplicon length, reference-database coverage, intragenomic variation, and taxonomic resolution can influence relative-abundance and species-level assignments (23). The original taxonomic assignments were generated using a proprietary, internally maintained provider database for which no formal version identifier, release date, or complete description of the underlying reference sources was available at the time of resubmission. Independent comparison of the principal ASVs against UNITE General FASTA v10.0 showed greater concordance at genus than at species level (79.1% versus 50.0% among provider-resolved assignments; Supplementary Table S3). This comparison supports interpretation of the broader genus-level patterns but also demonstrates that taxonomic resolution, particularly at species level, is database dependent. Species-level assignments are therefore treated as provisional, and pipeline-defined ASVs are interpreted as sequence features rather than independently confirmed fungal species. Because the UNITE comparison was a validation analysis rather than a complete reanalysis, the provider-generated taxonomic composition and downstream diversity outputs remain the primary results. Similarly, ITS2 qPCR values were retained as target copies per microliter. Conversion to genome equivalents would be unreliable because fungal rDNA copy number varies by more than two orders of magnitude among taxa (18).

Strengths of the study include standardized sampling during estrus, explicit documentation of the overlapping pool design, matched mare membership in the sampling-method pools, inclusion and sequence-resolved evaluation of laboratory controls, positive-control recovery, and integrated reporting of sequencing, qPCR, taxonomic, diversity, and conventional-culture findings. These features improve the transparency and descriptive value of the study but do not overcome the absence of biological replication resulting from the single-library-per-category pooled design. The main limitations are the absence of individually sequenced mares, one composite library per condition, overlap between the non-pregnant and sampling-method pools, absence of field and sampling blanks, detection of a low-abundance sequence feature shared by a negative control and two biological libraries, reliance on a proprietary taxonomic reference database for the primary provider-generated outputs despite independent comparison of the principal ASVs against UNITE, incomplete and mostly qualitative culture records, absence of cytology or histopathology, lack of inferential analysis, and inability of DNA sequencing to determine viability or uterine pathology. Future studies should sequence individual mares, prespecify sufficient biological replication, include and sequence field, sampling, extraction, and library-preparation controls, use publicly accessible and versioned fungal reference databases within the primary taxonomic analysis pipeline, apply standardized quantitative bacterial and mycological culture with complete paired sampling, integrate cytology or histopathology, and account for fungal rDNA copy-number variation.

## 5 Conclusion

Fungal ITS2 DNA was detected in all four pooled equine uterine sample libraries, with descriptive differences in taxonomic composition, pool-level diversity, ITS2 target concentration, and ordination position among the pregnancy-outcome and sampling-method libraries. Because each analytical category was represented by a single pooled sequencing library, these findings do not establish a pregnancy-associated fungal profile or agreement, equivalence, or superiority between LVL and double-guarded uterine swabbing. The absence of fungal growth under the applied culture conditions does not exclude viable fungi, while ITS2 detection alone does not demonstrate uterine colonization, inflammation, or infection. These findings are hypothesis-generating and require confirmation in individually sequenced mares using biological replication, comprehensive field and laboratory controls with prespecified control-informed filtering, and complementary microbiological culture, cytology, and histopathology.

## Data Availability

Raw paired-end ITS2 amplicon sequencing data for the four pooled biological libraries have been deposited in the NCBI Sequence Read Archive under BioProject accession number PRJNA1498564. The data will be released upon publication or automatically on 1 August 2027, whichever occurs first. Paired-end ITS2 FASTQ files for the extraction and library-preparation blanks, provider-supplied sequence-variant tables for the biological libraries and controls, and a summary of exact sequence overlap between control and biological-library ASVs are provided in Supplementary Data S1. Curated provider-generated outputs from the targeted sensitivity analysis excluding seq97 are provided in Supplementary Data S2. Individual mare-level metadata and the mapping between pooled-library labels, provider identifiers, BioSample accessions, SRA records, and biological-library FASTQ files are provided in Supplementary Tables S1 and S2, respectively. Results of the independent comparison of principal fungal ASVs against UNITE are provided in Supplementary Table S3. Additional data supporting the findings of this study are available from the corresponding author upon reasonable request.
