# Supplementary material for: Fungal DNA in pooled equine uterine samples: an exploratory ITS2 amplicon sequencing pilot study
Source: Front Vet Sci. 2026 Oct 27;13:1978506. doi: 10.3389/fvets.2026.1978506 (PMC13748243; doi:10.3389/fvets.2026.1978506)
Supplement: Supplementary file 3 [file Table_1.docx]

# Fungal DNA in Pooled Equine Uterine Samples: An Exploratory ITS2 Amplicon Sequencing Pilot Study

# Urtė Pelenė, Rasa Nainienė, Rūta Šveistienė, Inga Merkelytė, Artūras Šiukščius

**Supplementary Table S1.** Individual mare characteristics, reproductive status, sampling methods, pooled-library membership, and conventional culture findings for all 21 enrolled mares.

**A. Mare characteristics and study allocation**

| Mare ID | Age, years | Parity | Days postpartum | Breeding status | Pregnancy outcome | LVL collected | Swab collected | Pooled library membership | Reason not represented in sequencing |
| --- | --- | --- | --- | --- | --- | --- | --- | --- | --- |
| M01 | 11 | 3 | 812 | Not bred | Not applicable | Yes | No | None | Unbred; paired swab unavailable |
| M02 | 13 | 5 | 707 | Not bred | Not applicable | Yes | No | None | Unbred; paired swab unavailable |
| M03 | 9 | 3 | 403 | Not bred | Not applicable | Yes | No | None | Unbred; paired swab unavailable |
| M04 | 7 | 2 | 11 | Natural mating | Pregnant | Yes | No | Pregnant-LVL pool (1FR) | — |
| M05 | 15 | 7 | 791 | Natural mating | Non-pregnant | Yes | No | Non-pregnant-LVL pool (2NFR) | — |
| M06 | 7 | 2 | 55 | Natural mating | Pregnant | Yes | No | Pregnant-LVL pool (1FR) | — |
| M07 | 9 | 4 | 28 | Natural mating | Pregnant | Yes | No | Pregnant-LVL pool (1FR) | — |
| M08 | 9 | 3 | 28 | Natural mating | Pregnant | Yes | No | Pregnant-LVL pool (1FR) | — |
| M09 | 18 | 15 | 28 | Natural mating | Non-pregnant | Yes | No | Non-pregnant-LVL pool (2NFR) | — |
| M10 | 5 | 1 | 27 | Not bred | Not applicable | Yes | No | None | Unbred; paired swab unavailable |
| M11 | 9 | 2 | 1111 | Natural mating | Non-pregnant | Yes | No | Non-pregnant-LVL pool (2NFR) | — |
| M12 | 4 | 0 | Not applicable | Natural mating | Pregnant | Yes | No | Pregnant-LVL pool (1FR) | — |
| M13 | 7 | 2 | 421 | AI with frozen semen | Non-pregnant | No | Yes | None | Swab only; outcome pools LVL-based |
| M14 | 9 | 3 | 413 | AI with frozen semen | Pregnant | No | Yes | None | Swab only; outcome pools LVL-based |
| M15 | 11 | 4 | 59 | AI with frozen semen | Non-pregnant | No | Yes | None | Swab only; outcome pools LVL-based |
| M16 | 13 | 5 | 1169 | Natural mating | Non-pregnant | Yes | Yes | 2NFR; 3LVL; 4SWAB | — |
| M17 | 17 | 7 | 399 | Natural mating | Non-pregnant | Yes | Yes | 2NFR; 3LVL; 4SWAB | — |
| M18 | 11 | 4 | 1129 | Natural mating | Non-pregnant | Yes | Yes | 2NFR; 3LVL; 4SWAB | — |
| M19 | 8 | 2 | 45 | AI with frozen semen | Pregnant | No | Yes | None | Swab only; outcome pools LVL-based |
| M20 | 12 | 6 | 67 | Natural mating | Non-pregnant | Yes | Yes | 2NFR; 3LVL; 4SWAB | — |
| M21 | 7 | 2 | 55 | Not bred | Not applicable | Yes | Yes | 3LVL; 4SWAB | — |

The paired sampling-method component was initiated after samples had been collected from M01, M02, M03, and M10; therefore, corresponding swab samples were unavailable. M13, M14, M15, and M19 had swab samples only and were not included because the pregnancy-outcome sequencing comparison was restricted to LVL-derived samples.

**B. Individual conventional culture findings**

| Mare ID | LVL culture finding | Swab culture finding |
| --- | --- | --- |
| M01 | No pathogenic or potentially pathogenic bacteria isolated; no fungal growth | Not collected |
| M02 | No pathogenic or potentially pathogenic bacteria isolated; no fungal growth | Not collected |
| M03 | No pathogenic or potentially pathogenic bacteria isolated; no fungal growth | Not collected |
| M04 | *Streptococcus equi* subsp. *zooepidemicus* and *Streptococcus mitis*; no fungal growth | Not collected |
| M05 | No pathogenic or potentially pathogenic bacteria isolated; no fungal growth | Not collected |
| M06 | No pathogenic or potentially pathogenic bacteria isolated; no fungal growth | Not collected |
| M07 | No pathogenic or potentially pathogenic bacteria isolated; no fungal growth | Not collected |
| M08 | No pathogenic or potentially pathogenic bacteria isolated; no fungal growth | Not collected |
| M09 | No pathogenic or potentially pathogenic bacteria isolated; no fungal growth | Not collected |
| M10 | No pathogenic or potentially pathogenic bacteria isolated; no fungal growth | Not collected |
| M11 | *Streptococcus equi* subsp. *zooepidemicus* and *Staphylococcus equorum*; no fungal growth | Not collected |
| M12 | No pathogenic or potentially pathogenic bacteria isolated; no fungal growth | Not collected |
| M13 | Not collected | No pathogenic or potentially pathogenic bacteria isolated; no fungal growth |
| M14 | Not collected | No pathogenic or potentially pathogenic bacteria isolated; no fungal growth |
| M15 | Not collected | *Streptococcus equi* subsp. *zooepidemicus*, one colony; no fungal growth |
| M16 | *Microbacterium maritypicum*; no fungal growth | *Staphylococcus capitis* and *Corynebacterium uterequi*; no fungal growth |
| M17 | Not cultured | *Staphylococcus equorum*, one colony; no fungal growth |
| M18 | Not cultured | *Staphylococcus equorum*, reported in a very low quantity; no fungal growth |
| M19 | Not collected | *Actinomyces urogenitalis*; no fungal growth |
| M20 | *Globicatella sanguinis*, *Staphylococcus capitis*, and *Achromobacter insolitus*; no fungal growth | *Corynebacterium uterequi* and *Staphylococcus equorum*, reported in a low quantity; no fungal growth |
| M21 | *Corynebacterium uterequi*, *Paraburkholderia xenovorans*, and *Streptococcus equi* subsp. *zooepidemicus*; no fungal growth | *Corynebacterium uterequi* and *Streptococcus equi* subsp. *zooepidemicus*; no fungal growth |

**Abbreviations:** AI, artificial insemination; LVL, low-volume uterine lavage; SWAB, double-guarded uterine swab. The 1FR and 2NFR biological pools comprised LVL samples from pregnant and non-pregnant mares, respectively. The 3LVL and 4SWAB biological pools comprised paired LVL and swab samples from the same five mares. Each biological pool generated one sequencing library. Four mares contributed samples to 2NFR, 3LVL, and 4SWAB; therefore, library memberships overlap and should not be summed. “Not collected” indicates that the corresponding sample type was not obtained, whereas “not cultured” indicates that the sample was collected but conventional culture was not performed. Pregnancy outcome and days postpartum were recorded as “not applicable” for unbred and nulliparous mares, respectively. Culture findings are reported as documented in the available diagnostic laboratory records.
