# Supplementary material for: Fungal DNA in pooled equine uterine samples: an exploratory ITS2 amplicon sequencing pilot study
Source: Front Vet Sci. 2026 Oct 27;13:1978506. doi: 10.3389/fvets.2026.1978506 (PMC13748243; doi:10.3389/fvets.2026.1978506)
Supplement: Supplementary file 4 [file Table_2.docx]

# Fungal DNA in Pooled Equine Uterine Samples: An Exploratory ITS2 Amplicon Sequencing Pilot Study

# Urtė Pelenė, Rasa Nainienė, Rūta Šveistienė, Inga Merkelytė, Artūras Šiukščius

**Supplementary Table S2.** Mapping of pooled-library labels, sequencing-provider sample identifiers, BioSample accessions, Sequence Read Archive records, and deposited FASTQ files.

| Pooled-library label | Provider sample ID | BioSample accession | SRA run accession | SRA record title | Forward-read FASTQ file | Reverse-read FASTQ file |
| --- | --- | --- | --- | --- | --- | --- |
| Pregnant-LVL pool (1FR) | zr23311-1 | SAMN61848694 | SRR39749265 | Pregnant mares pooled LVL ITS2 | zr23311_1ITS2_R1.fastq.gz | zr23311_1ITS2_R2.fastq.gz |
| Non-pregnant-LVL pool (2NFR) | zr23311-2 | SAMN61848695 | SRR39749264 | Non-pregnant mares pooled LVL ITS2 | zr23311_2ITS2_R1.fastq.gz | zr23311_2ITS2_R2.fastq.gz |
| Paired LVL pool (3LVL) | zr23311-3 | SAMN61848696 | SRR39749263 | Combined pooled LVL ITS2 | zr23311_3ITS2_R1.fastq.gz | zr23311_3ITS2_R2.fastq.gz |
| Paired swab pool (4SWAB) | zr23311-4 | SAMN61848697 | SRR39749262 | Combined pooled uterine double-guarded swab ITS2 | zr23311_4ITS2_R1.fastq.gz | zr23311_4ITS2_R2.fastq.gz |

All records belong to BioProject PRJNA1498564 (submission SUB16346185). The records are scheduled for public release upon publication or automatically on 1 August 2027, whichever occurs first. Each row represents one pooled biological sample and its corresponding paired-end sequencing library. LVL, low-volume uterine lavage; SRA, Sequence Read Archive.
